# On Infanticide

**Published:** 1820-06

**Authors:** W. Hutchinson

**Affiliations:** Sackville-street


THE LONDON

### Medical and Physical Journal.

6 OF VOL. XLIII.] JUNE, 1820. [NO. 256.

"For many fortunate discoveries in medicine, and for the detection of numerous "errors, the world is indebted to the rapid circulation of Monthly Journals; "and there never existed any work to which the Faculty in EUROPE and "AMERICA were under deeper obligations than to the Medical and Physical "Journal of London, now forming a long, but an invaluable, series." RUSH.

### Original Communications, Select Observations, etc.

### MEDICAL JURISPRUDENCE.

### DISSERTATION II.—*On Infanticide.*

[In continuation from page 361.]

IN order to attain the last object of medical inquiry in the case under consideration,—the knowledge of the probable relation of the body of the discovered child to the suspected mother,—it is in the first instance requisite to form a decision respecting the length of time that has elapsed since the birth of the child. It will be necessary to consider here, the period which the infant may have lived, and that which has passed since its death. The state of the navel is, in general, the best mean for determining the former point. The umbilical cord separates from the navel, ordinarily, about the fifth day, and is almost always partially detached on the fourth; the ulcerated surface is commonly healed by the eighth or ninth day. These two circumstances, and the degree of the process towards them, will enable us to determine this part of the question with sufficient precision in the generality of cases. The state of the lungs, the canalis arteriosus, and umbilical vessels, will also furnish additional useful evidence, on the grounds which have been designated in several parts of this dissertation. The other point is often one of great difficulty, as the changes which take place in the body after death are so considerably modified by numerous external circumstances: thus, putrefaction takes place more rapidly in summer than in winter; and during a southern wind, and in a moist situation, than when the place is dry and the wind northerly. It putrefies quickly in stagnant water exposed to the rays of the sun: but, in a running stream, and in a cold season, it is often preserved without sensible decomposition for a long time, and is sometimes converted into *adipocire*. It is also preserved, apparently fresh, for a long period in a marlous or argillaceous soil; and, as it would appear from some observations, in *cloacas* developing an abundance of gases. A body exposed in the open air in a dry and cold winter will be preserved without much evident decomposition for many weeks. But, though the body may be preserved apparently fresh for a long time in a dry and cold wind, a shrivelled and dried state of it, especially of the globe of the eye and the tendons of the muscles, will often clearly indicate, to an accurate observer, that it has long been exposed in this way.

The value of the determination of the above matters, consists in the application of the evidence in question to the period which has apparently elapsed since the delivery of the suspected mother. The medical practitioner is generally required to examine the mother, and give his opinion on this point, even though it may be known, with moral certainty, from various circumstances in her conduct.

The more strongly-marked and characteristic signs of recent delivery at or near the full term of utero-gestation, and before above three or four days have elapsed since its occurrence, are, a slight paleness of the face; the eye is a little sunken, and surrounded by a purplish or dark-brown coloured ring. The pulse is full and undulating; the skin soft, supple, rather warmer than ordinary, and covered with a moisture having a peculiar and somewhat acid odour. The breasts are tumid, and, on pressure, emit a lactiform fluid; there is a dark areola round the nipples. The belly is soft; the skin of the abdomen is lax, lies in folds, and is traversed in various directions with shining reddish and whitish lines, and which especially extend from the groins and the pubis towards the navel. A line of a brownish colour commonly extends from the centre of the pubis to the navel; and, when the point of the finger is run along this, a separation of the muscular fibres parallel to the median line is evident, and which is broadest towards the navel. The uterus may be felt through the abdominal parietes, voluminous, firm, globular, rising very near as high as the umbilicus, contracting and expanding under the hand, on pressure. A discharge of serous fluids mingled with blood, of a peculiar acid odour, takes place from the vagina, and this fluid often contains small clots of blood. The external genital organs, that is to say, the labia pudendi and vagina, are tumefied, and dilated throughout the whole of their extent: the orifice of the uterus is soft, supple, and considerably dilated. Sometimes the anterior margin of the perineum is a little torn, or it is lax, and appears to have suffered considerable distension. Professor Chaussier states, that no disease or affection besides parturition can produce the whole series of signs just described. After the fourth or fifth day, they become considerably less evident; and, at the end of seven or eight days, but few or none of them can be observed. The secretion of milk may continue; but Professor John Burns (who is here quoted as the latest good author on this subject), says, "it is possible for this secretion to take place independently of pregnancy."*

It may happen that the practitioner will be required to form an analogous judgment from examination of the dead body of the mother: the appearances generally observable on this occasion are thus described by Professor Burns.†

"If the woman die of hemorrhage, or from any cause destroying her, soon after her delivery, the uterus is found like a large flattened pouch, from nine to twelve inches long. The cavity contains coagula or a bloody fluid, and its surface is covered by the remains of the decidua. Often the marks of the attachment of the placenta are very visible. This part is of a dark colour, so that the uterus is thought to be gangrenous by those who are not aware of the circumstance. The surface being cleaned, the sound substance of the womb is seen. The vessels are extremely large and numerous. The fallopian tubes, round ligaments, and surface of the ovaria, are so vascular, that they have a purple colour. The spot where the ovum escaped, is more vascular than the rest of the ovarian surface. This state of the uterine appendages continues until the womb has returned to its unimpregnated state.

"A week after delivery, the womb is as large as two fists. At the end of a fortnight, it will be found about six inches long, generally lying obliquely to one side. The inner surface is still bloody, and covered partially with a pulpy substance, like decidua. The muscularity is distinct, and the orbicular direction of the fibres round the orifice of the tubes very evident. The substance is whitish. The intestines have not yet assumed the same order as usual, but the distended coecum is often more prominent than the rest.

"It is a month at least before the uterus returns to its natural state; but the os uteri rarely, if ever, closes to the same degree as in the virgin state."

The consideration of the appearances just described may become a matter of serious importance on another occasion, besides that just indicated, as when a woman is suspected of having produced a child which cannot be found; for, though the coroner cannot take an inquest in this case, the affair may be inquired into by the justices of the peace, and brought into a court of criminal judicature.

---

* *Principles of Midwifery*, page 451, fourth edition.

† *Op. cit.* p. 452.

The medical practitioner has now only to present his formal report, containing his conclusions in respect to the stated objects of this inquiry, in order that his duties may be completely fulfilled on this occasion; and, when he has proceeded in the manner indicated in this dissertation, he will have arrived so near to the requisite conclusions, that but little further reflection will be necessary to enable him to form his judgment in a satisfactory manner. The order of his notes, too, is that which is proper for him to adopt in the construction of his report.

After having concisely related the time and the circumstances under which he was required to visit the body, and the observations he may have made in conformity to the directions given on a former occasion, he will state the results of the several series of researches described in this dissertation, as far as the subjects of them have been observed or examined. When wounds or other consequences of violence are present, he should describe particularly their characters, and the mode in which the body has been interested by them; and this should be done in the manner of a simple demonstration. His rational inferences should follow the complete exposition of the evidence whence they are drawn. His judgments should be advanced in the most precise and simple manner. He should not enter into any arguments or apologies for his opinions; neither should he attempt to give additional weight to them, by adducing those of any writer on the subject, as the propriety of the application of these to the case in question will not be recognized by the court. He is supposed by the legal authorities to be sufficiently acquainted with the physiological laws which relate to the subject under consideration; and it is his own interpretation of what he has observed that is here required.

The foregoing remarks relate expressly to the mode in which the report should be given; but, should he be personally examined by the court, and the grounds for his judgments enquired into, it will be prudent in him to support his arguments respecting certain points by the opinions of some other estimable persons or written authorities. For example, such as relate to the possibility of the respiration of a child before it is completely born; the experiments on which the determination of the value, and the proper application, of the pulmonary tests have been founded; observations on several accidents which may occur during parturition; the general effects of drowning; and the like. He may also, on this occasion, adduce his reflections and opinions on such points of the moral qualities and conduct of the mother as come expressly under the cognizance of the medical practitioner: for instances, on the possibility of her being with child without being conscious of the fact; and on her ability to afford the necessary succours to the infant after its birth.

The destruction of the life of a child during the period of its existence regarded in the foregoing views, is not in itself contemplated distinctly from *homicide* at any other epoch of life by the laws of England. But some statutes have been enacted, at different times, respecting the commission of this crime under certain circumstances; as, where the subject is born a bastard: and it would indeed appear that the legislature have not considered it probable that such a crime could be perpetrated, at least by the mother, in any other case than that just mentioned; for it is to that alone all the peculiar provisions on this subject expressly relate.

The murder of bastard children by the mother was considered as a crime so difficult to be proved a century or two since, when physiological knowledge was in a very rude state, that a special legislative provision was made for its prevention by a statute passed in the reign of James the First, (xxi. c. 27.) which required, that any such mother endeavouring to conceal the death of the child, should prove, by one witness at least, that the child was actually born dead. This severe law, which made the concealment of the death almost conclusive evidence of the child's being murdered, was repealed, together with an Irish act upon the same subject, by a late statute,* which provides, "that the trials in England and Ireland, respectively, of women charged with the murder of any issue of their bodies, male or female, which, being born alive, would by law be bastard, shall proceed and be governed by such and the like rules of evidence, and of presumption, as are by law used and allowed to take place in respect to other trials for murder, and as if the said two several acts had never been made." The statute further provides, that the jury, if they acquit the prisoner of murder, may find that she was delivered of a bastard child, and endeavoured to conceal the birth; whereon the court may adjudge her to be committed to prison for any time not exceeding two years.

The actual perpetration of destructive violence is not absolutely necessary, in order to constitute this crime, any more than it is in respect to homicide under certain other circumstances: for, if the mother omit such care and aid as would be necessary to sustain the life of the infant, or expose the infant, or place it in such a situation as that death occurs in consequence of such act or of such want of aid and care, it would be murder by the common law, if a jury were satisfied that the mother contemplated, or could not in nature and reason but

* 43 Geo. III. c. 58, § 3.

have contemplated, the consequences which must have resulted from her act.

By the law of Scotland, the penalty of death is attached to the crime of infanticide, and in former times this penalty might be put in force on merely presumptive evidence; for the statute of 1690 (c. 21.) enacts, that "any woman who shall conceal her being with child during the whole time of her pregnancy, and shall not call for, or make use of, help in the time of birth, is to be reputed the murderer, if the child be found dead or amissing."

The latest conviction on this statute occurred in the year 1776. Since then, until the enactment of a recent one, in cases where the charge had been upon the *statute only*, and which have otherwise been favourable to the delinquent, it had been common for the prosecutor to consent to the delinquent's petition, praying to be banished forth of Scotland, or for other arbitrary punishment. To correct this, as it was deemed, too lenient course of practice, and yet avoid recurring to the rigour of the act of 1690, it has more lately been thought advisable to repeal that statute, and to substitute in its stead a qualified and more temperate enactment; under which, if a woman "shall conceal her being with child during the whole period of her pregnancy, and shall not call for, or make use of, help or assistance in the birth; and if the child shall be found dead or amissing; the mother, being lawfully convicted thereof, shall be imprisoned for a period not exceeding two years."*

The fundamental circumstances required by the above statute for the attachment of the penalty it enacts, are, that the woman be proved to have been pregnant, and that the child be missing, or that the dead body of a child be found, which is proved to be her child. In the former case, the proof of the pregnancy can only be by those signs in the state of the woman's person which show her to have been recently delivered. The statute has presumed that decisive evidence of pregnancy may in this way be obtained, else it could not have ordered any thing in the case of a child that is missing; and that, where the birth is a mere abortion, or where criminal violence has not been inflicted on it, it will not be concealed. In the case of the body being found, the above-mentioned evidence may be strengthened, if the woman, on being shown the body, has admitted it to be that of her child; which confession, of itself, has always been held sufficient proof of that material fact against her.

David Hume, in his commentaries on this statute, remarks, that it is clear, from the expressions and context, that it is not necessary to prove that the child was born at the full time, or

---

* 9 Geo. III. c. 14.

that it was born living, in order that the penalty of it may be put in force; because neither of these circumstances can be determined in the case of the child being missing. But, in case of the child being found, those circumstances become of serious importance in regard to the new statute, where imprisonment is the only punishment for the delinquency enacted in the statute in question. The statute rests on presumptive proof of guiltiness, on the part of the mother, of the murder of the child in the case alluded to in it: therefore, if the child be found, and it appear to have been dead born, she may avoid the penalty of the statute. In a word, the presumptive proof on which the statute authorizes conviction, is liable to be outweighed by evidence of such things as are naturally exclusive of guilt.

The crime of simple *exposure* of infant children is punished by an arbitrary penalty, which does not materially differ from that attached to it by the English statute law under similar circumstances: the nature of the delinquency being contemplated in the same manner by the legal institutes, and similar rules of moral and presumptive evidence directing the juridical verdict in both nations.* W. HUTCHINSON.

*Sackville-street; May 1st*, 1820.

---

* The laws of Scotland respecting *Feticide* are here introduced, having been omitted in the dissertation on that subject.

According to the law of Scotland, it is necessary, in order to constitute *homicide*, that the slaughter be of a person or existing human creature: therefore, all procuring of abortion, or destruction of a fetus, whether quick or not, is excluded; because, though it be quick, still it is only *pars viscerum matris*, and not a separate being, or such of which it can with certainty be said whether it would have become a quick birth or not.* It is however true, that, on the 10th November, 1606, Patrick Deanes had sentence of death for the slaughter of his wife and the child in her womb. As also, on the 12th February, 1631, Thomas Davison and Effie Gibb received the like sentence, for the murder of Elizabeth White, Davison's wife, "and the bairn in her belly being near to the full time." Again, there is the case of Patrick Robertson and Marion Kempt, for notorious adultery and taking of a "*poisonable draught*," wherewith she destroyed the child in her womb. But, in all these instances, HUME remarks, "another and a capital crime concurred with the destruction of the child; and it cannot be known, from the short and general expressions of the record, that the latter was found relevant as a murder by itself: neither have I found any instance of that description in later times." He thinks there may be room for argument against the delinquent in the case of a child which is born alive, but dies immediately upon the birth, in consequence of evil medicines which have previously been administered to the mother: as HAWKINS (i. 31, No. 16.) had suggested respecting the laws of England at the time he wrote. But, he properly adds, "it is difficult to imagine that in these circumstances a decisive proof shall ever be obtained of the true cause of the death of the child." He further remarks on this subject,† "If a man administer a potion to a woman without her knowledge, in order to procure abortion; and if the composition be of that powerful nature, as probably it will, to be attended with a plain risk of life to the person who takes it, especially when it is taken in this unguarded manner; and if the woman in consequence die; this seems to be nothing less than murder: because he shows the same disregard of

* *Commentaries on the Laws of Scotland.* By David Hume, Esq. Professor of the Law of Scotland in the University of Edinburgh. Vol. I. p. 274.

† *Opera citata*, vol. i. p. 407.

her life and safety, and exposes her to the same risk, as by doing outward violence to her person. A cause of this sort was tried at Aberdeen on the 10th May, 1785; the case of Robert Dalrymple, a flax-dresser, and Robert Joyner, a druggist. The fact, as alleged, seems to have been, that these persons, having each of them a young woman with child to him, they administered some violent drug to them without their knowledge, (as was supposed for the purpose of procuring abortion,) in consequence of which both the women died in the course of the same night. The libel was laid, without any mention of the supposed purpose of the prescription, as for murder by poison, with an alternative of culpable homicide. The court found it relevant to infer the pains of law. Joyner was *outlawed;* and the libel was found *not proven* against Dalrymple."

Although the penalty attached to homicide is not found relevant in cases of simple feticide, there is a penalty applied to it, and which, it appears from a case related by Hume, is arbitrary. He says, "Libel was found relevant (24th, 25th, and 28th July, 1806,) to infer an arbitrary punishment, in the case of Catharine Robertson and George Batchelor, for the crime of "wilful causing or procuring a pregnant woman to abort, or to part in an untimely manner with the *fetus* or child in her womb." It related, that this object was accomplished by means of some instrument made use of upon her body, which brought on a premature labour, and she was delivered in the fifth or sixth month of her pregnancy. They were convicted, and had sentence of transportation for seven years.*

* Hume's *Supplemental Notes to his Commentaries on the Law of Scotland respecting Crimes* p. 62.

